# Mutant p53 reactivation restricts the protumorigenic consequences of wild type p53 loss of heterozygosity in Li-Fraumeni syndrome patient-derived fibroblasts

**DOI:** 10.1038/s41418-024-01307-4

**Published:** 2024-05-14

**Authors:** Himanshi Agarwal, Perry Tal, Naomi Goldfinger, Esita Chattopadhyay, David Malkin, Varda Rotter, Ayush Attery

**Affiliations:** 1 Department of Molecular Cell Biology, Weizmann Institute of Science, Rehovot, Israel; 2 Department of Genetics and Genome Biology and the Division of Hematology/Oncology, The Hospital for Sick Children, Toronto, ON Canada; 3 Departments of Medical Biophysics and Pediatrics, University of Toronto, Toronto, ON Canada; 4 Present Address: Department of Tumor Cell Biology, St Jude Children’s Research Hospital, Memphis, TN USA

**Keywords:** Tumour-suppressor proteins, Tumour-suppressor proteins

## Abstract

The p53 tumor suppressor, encoded by the *TP53* gene, serves as a major barrier against malignant transformation. Patients with Li-Fraumeni syndrome (LFS) inherit a mutated *TP53* allele from one parent and a wild-type *TP53* allele from the other. Subsequently, the wild-type allele is lost and only the mutant *TP53* allele remains. This process, which is termed loss of heterozygosity (LOH), results in only mutant p53 protein expression. We used primary dermal fibroblasts from LFS patients carrying the hotspot p53 gain-of-function pathogenic variant, R248Q to study the LOH process and characterize alterations in various pathways before and after LOH. We previously described the derivation of mutant p53 reactivating peptides, designated pCAPs (p53 Conformation Activating Peptides). In this study, we tested the effect of lead peptide pCAP-250 on LOH and on its associated cellular changes. We report that treatment of LFS fibroblasts with pCAP-250 prevents the accumulation of mutant p53 protein, inhibits LOH, and alleviates its cellular consequences. Furthermore, prolonged treatment with pCAP-250 significantly reduces DNA damage and restores long-term genomic stability. pCAPs may thus be contemplated as a potential preventive treatment to prevent or delay early onset cancer in carriers of mutant p53.

## Introduction

*TP53* is a tumor suppressor gene that encodes the p53 protein. Pathogenic variants (PVs) in *TP53* are found in nearly 50% of all human cancers, with most PVs occurring within the DNA-binding domain of p53 [1, 2] and resulting in highly stabilized mutant p53 (mutp53) proteins that not only lose their tumor suppressor activity but often even acquire oncogenic gain-of-function. p53 can regulate metabolism, and cancer cells expressing mutp53 display increased mitochondrial metabolism [3]. p53 stabilization and activation is induced by a variety of cellular stressors, including ionizing radiation [4], UV radiation [5], and ribonucleotide depletion [6]. p53 is also implicated in DNA repair [7, 8], and affects homologous recombination, nonhomologous end-joining, mismatch repair, and nucleotide-excision repair and base-excision repair [9–12]. The p53 protein transcriptionally activates many DNA-damage response genes, such as *XPC*, *FEN1*, *DDB2*, *Mlh1*, and *Msh2* [13–17]. If the damage is beyond repair, p53 induces apoptosis [18]. In the absence of functional p53, DNA damage is devolved to daughter cells, resulting in genomic instability.

Patients with the rare cancer predisposition Li-Fraumeni syndrome (LFS), carrying germline heterozygous *TP53* PVs, appear to exhibit normal development yet are at an almost 100% lifetime risk to develop a wide spectrum of cancers [19, 20], predominantly sarcomas, adrenocortical carcinomas, brain tumors, breast cancer and leukemias [21], with a greater than 83-fold increased risk to develop multiple primary malignancies [22]. The emergence of cancer in *TP53 PV* carriers is often associated with the loss of the wild-type (WT) *TP53* allele, retaining only the mutant allele in the cancer cells. This process is referred to as p53 loss of heterozygosity (p53 LOH). WT p53 LOH is often characterized by DNA shuffling and breakage, leading to chromothripsis [23, 24]. Since mutp53 cannot induce the expression of MDM2, the key negative regulator of p53 stability, mutp53 accumulates in the cells. *TP53* LOH is considered a prerequisite for mutp53 stabilization and gain-of-function. LOH of p53 is a critical event in cancer development, as it allows cells to bypass important regulatory mechanisms and promotes cancer progression. R248Q is one of the most common hotspot mutations in the DNA-binding domain of p53. LFS patients carrying the R248Q mutation exhibit markedly accelerated tumor onset (10.5 years earlier) and more frequent tumors per individual than LFS patients carrying loss-of-function *TP53* mutations. *TP53* LOH is observed in 40–60% of their tumors.

Currently, there are no cancer-preventive measures for LFS mutation carriers, and individuals with a family history of LFS undergo intensive clinical surveillance [25], to facilitate early detection and diagnosis of cancer. We previously reported the identification of a group of small peptides, called p53 conformation–activating peptides (pCAPs), which bind p53 and stabilize its structure, leading to reactivation of mutp53 into a functional protein capable of transcriptional transactivation of p53 target genes and execution of programed cell death of cancer cells expressing mutp53 [26]. Here, we characterize protumorigenic consequences of WT *TP53* LOH in LFS cells and report that these consequences can be abolished by pCAP-mediated mutant p53 reactivation, suggesting that pCAPs may be considered for cancer prevention in LFS mutation carriers.

## Materials and methods

### Mice

Female NOD.CB17-prkdc-SCID/NCrHsd mice aged 6 weeks (Harlan, Rehovot, Israel) were used in this study. Animal protocols were approved by the Institutional Animal Care and Use Committee of the Weizmann Institute of Science.

### Cell culture

Skin biopsy samples were cut into small pieces, and incubated with collagenase in a 37 °C incubator for 1.45 h. Samples were then centrifuged at 370 × *g* for 10 min and the supernatant was removed. Trypsin/EDTA was added to the pellet, and cells were pipetted for homogeneity and incubated at 37 °C for 30 min. The cells were then centrifuged, washed with 1xphosphate buffer saline and plated in alpha-MEM and 20% fetal calf serum (FCS), and subsequently maintained at 37 °C in Dulbecco’s modified eagle’s medium (DMEM; Biological Industries, Bet-Haemek, Israel) supplemented with 10% FCS 60 mg/mL penicillin, and 100 mg/mL streptomycin, in a humidified atmosphere of 5% CO_2_. The cells in culture were checked for mycoplasma contamination.

“Early passage” cells refers to passages 1-12; “medium passage” refers to passages 13–28, and “late passage” refers to passages 29 and later.

### RNA analysis

Total RNA was isolated by using a Nucleospin II kit (Macherey-Nagel, Duren, Germany), according to the manufacturer’s instructions. RNA (1 µg) was reverse-transcribed using Bio-RT (Bio-Lab, Jerusalem, Israel) and random hexamer plus oligo-dT primers (New England Biolabs, Ipswich, MA, USA). cDNA (100 ng) was sequenced at the sequencing unit by using a p53 DNA-binding domain–specific primer (Table 1).

**Table 1:** Primers for cDNA sequencing of p53 gene.

Primers	Sequence (5′-3′)
Full length forward p53	CCCCTCTGAGTCAGGAAACA
Full length reverse p53	CAGTCTGAGTCAGGCCCTTC
Sequencing primer	GTGCAGCTGTGGGTTGATT

### Measurement of intracellular ATP levels

Fibroblasts were seeded (18,000 cells/well) in a 96-well plate. After 24 h, cells were lysed by boiling in tris-EDTA solution (pH 7.75). Intracellular ATP was measured using a luciferin/luciferase-based assay (ATP Bioluminiscence Assay Kit CLS II; Roche, Germany), following the manufacturer’s instructions.

### Transmission electron microscopy

Cells were fixed with 3% paraformaldehyde and 2% glutaraldehyde in 0.1 M cacodylate buffer containing 5 mM CaCl_2_ (pH 7.4), and then in 1% osmium tetroxide supplemented with 0.5% potassium hexacyanoferrate trihydrate and potassium dichromate in 0.1 M cacodylate (1 h), stained with 2% uranyl acetate in water (1 h), dehydrated in graded ethanol solutions, and embedded in Agar 100 epoxy resin (Agar Scientific Ltd., Stansted, UK). Ultrathin sections (70-90 nm) were viewed and photographed with an FEI Tecnai SPIRIT (FEI, Eindhoven, Netherlands) transmission electron microscope (TEM) operated at 120 kV and equipped with an EAGLE CCD camera. TEM was performed by the electron microscopy core facility of the Weizmann Institute of Science.

### “Scratch” assay

Fibroblasts (1 × 10^6^) at early and late passages were grown in serum-free, phenol-red-free DMEM for 48 h in a 10 cm dish. Conditioned medium (CM) from early and late passage cells was collected and briefly centrifuged at 250 × *g* before being used for the wound-healing assay. U2OS cells (ATCC) were seeded at 20,000 cells/well in a 96-well plate and maintained in normal DMEM supplemented with 10% FCS. After 6 h, a scratch was made, and cells were washed with phosphate-buffered saline (PBS). CM(50 µL) was added to each well and the plate was placed in a humidified CO_2_ incubator at 37 °C. After 16 h, the CM was replaced by DMEM with 10% FCS, and cells were imaged at 0, 4, 8, 16, and 24 h. Data were quantified with ImageJ software.

### Lactate assay

Fibroblasts (1 × 10^6^) were grown in serum-free, phenol-red-free medium for 48 h and conditioned medium (CM) was collected after centrifugation of the cells at 250 × *g* for 5 min. The CM was used for measuring extracellular lactate concentration with Sigma-Aldrich lactate assay kit (catalog number MAK064), following the manufacturer’s instructions.

### Senescence assay

Cellular senescence was monitored by a β-galactosidase assay (Sigma-Aldrich #CS0030). Briefly, cells were washed with PBS, fixed for 5–10 min at room temperature, washed thrice with PBS and stained with staining solution overnight in a 37 °C incubator without CO_2_, and imaged by brightfield microscopy. The percentage of blue cells out of the total cells was quantified by ImageJ.

### Growth curve analysis

LFS fibroblasts (10^5^/10 cm dish) at the indicated passages were allowed to grow for 72 h in a humidified CO2 incubator in the presence of pCAP-250, scrambled peptide, or no peptide. Cells were then photographed in a brightfield microscope (3 different fields per sample). The number of cells in the treated cultures was normalized against the untreated culture, taken as 100%.

### Reverse transcriptase quantitative PCR (RT-qPCR)

Total RNA from cells was isolated using the NucleoSpin RNA II kit (Macherey-Nagel). A 1-μg sample of total RNA was reverse-transcribed into cDNA by using Bio-RT (Bio-Lab), deoxynucleoside triphosphates and random hexamer primers. RT-qPCR was performed on a Step One Plus instrument (Applied Biosystems, Grand Island, NY, USA) using SYBR Green PCR Master Mix (Quanta BioSciences, Gaithersburg, MD, USA). Values for specific genes were normalized to the *HPRT* housekeeping gene by using the ΔΔCt method. The tables with specific primer sequences are given in Tables 2, 3, and 4.

**Table 2:** RT-PCR primers for mitochondrial RT-PCR.

Primer name	Sequence (5′-3′)
h-ND1-Fwd	CACTAGCAGAGACCAACCGA
h-ND1-Rev	AGGGGAGAGTGCGTCATATG
h-ND2-Fwd	TCCAGCACCACGACCCTACT
h-ND2-Rev	TGATGGTGGGGATGATGAGGC
h-ND3-Fwd	ACCACAACTCAACGGCTACA
h-ND3-Rev	GTAGGGGTAAAAGGAGGGCA
h-ND4-Fwd	CCTTGGCTATCATCACCCGA
h-ND4-Rev	TCTTGGGCAGTGAGAGTGAG
h-ND4L Fwd	TCGCTCACACCTCATATCCTC
h-ND4L Rev	GGCCATATGTGTTGGAGATTG
h-ND5-Fwd	TAGGCGCTATCACCACTCTG
h-ND5-Rev	TGGACCCGGAGCACATAAAT
h-ND6 Fwd	GGGTGGTGGTTGTGGTAAAC
h-ND6 Rev	CCCCGAGCAATCTCAATTAC
h-COI-Fwd	GCCCACTTCCACTATGTCCT
h-COI-Rev	TGTATGCATCGGGGTAGTCC
h-COII Fwd	GGCCACCAATGGTACTGAAC
h-COII Rev	CGGGAATTGCATCTGTTTTT
h-COIII-Fwd	GTAAAACCCAGCCCATGACC
h-COIII-Rev	GTGGCCTTGGTATGTGCTTT
h-CYTB-Fwd	TGCCTCTTCCTACACATCGG
h-CYTB-Rev	GGGTGGGACTGTCTACTGAG
h-ATP6-Fwd	TAACCATACACAACACTAAAGGACGA
h-ATP6-Rev	GGGCATTTTTAATCTTAGAGCGAAA
h-ATP8 Fwd	TGGCCCACCATAATTACCC
h-ATP8 Rev	GCAATGAATGAAGCGAACAG

**Table 3:** RT-PCR primers for DNA damage genes.

Primers	Sequence (5′-3′)
h-XPC-Fwd	TTGTCGTGGAGAAGCGGTCTAC
h-XPC-Rev	CTTCTCCAAGCCTCACCACTCT
h-MSH2-Fwd	TTGGACCAAAGGAATGTGTT
h-MSH2-Rev	TCAGAATTCCTCCTCTTTGAAT
h-FEN1-Fwd	CGGGCTGTGGACCTCATC
h-FEN1-Rev	TCAAGTCGCCGCACGAT

**Table 4:** RT-PCR primers for p53 targets.

Primers	Sequence (5′-3′)
h-p21-Fwd	GGCAGACCAGCATGACAGATT
h-p21-Rev	GCGGATTAGGGCTTCCTCTT
h-PUMA-Fwd	GACCTCAACGCACAGTACGAG
h- PUMA -Rev	AGGAGTCCCATGATGAGATTGT
h-GDF15-Fwd	GACCCTCAGAGTTGCACTCC
h- GDF15-Rev	GCCTGGTTAGCAGGTCCTC
h-GAPDH-Fwd	ACCCACTCCTCCACCTTTGA
h-GAPDH-Rev	CTGTTGCTGTAGCCAAATTCGT

### Western blot analysis

Cell pellets were lysed in TLB buffer (50 mM Tris-HCl, 100 mM NaCl, 1% TritonX-100, 0.5% sodium deoxycholate, 0.1% SDS) supplemented with protease inhibitor cocktail (Sigma-Aldrich) for 15 min on ice and centrifuged at 10,000 ×*g* for 15 min. Supernatants were analyzed for protein concentration using BCA reagent (Thermo-Scientific, Grand Island, NY, USA). 50- or 80-μg protein extracts were boiled and loaded on 10%-12% SDS-polyacrylamide gel. Proteins were transferred to a nitrocellulose membrane at semi-dry conditions. Membranes were blocked using 5% dry milk in PBST. The following primary antibodies were used: DO-1 p53, β-actin (Santa Cruz Biotechnology, Dallas, TX USA; sc-47778), mutant p53 (Y5; Abcam, Cambridge, UK). Membranes were subjected to ECL Western blotting detection (Thermo Scientific) followed by analysis in ChemiDoc MP (Bio-Rad, Hercules, CA, USA).

### Immunofluorescence imaging

Fibroblasts were fixed in 4% paraformaldehyde, rinsed thrice with 1× PBS, washed twice with 0.1%TritonX-100 for 5 min each, once with 1× PBS supplemented with 0.2% Tween-20, and blocked overnight with 5% bovine serum albumin. Subsequently, the cells were stained with the indicated primary antibodies at room temperature for 1 h in a humidified chamber and then Incubated with secondary antibody under similar conditions. At least 100 cells were analyzed for all immunofluorescence experiments. Slides were imaged in a Zeiss 710 Meta system with 63×/1.4 oil immersion or 40×/0.95 Corr objective. The lasers used were Argon 488 nm (for FITC), DPSS 561 nm (for Cy3), and DPSS 405 nm (for DAPI). Zen software was used for image acquisition, and Image J software was used for quantification. Antibodies used were Anti-CPD (Cosmo Bio, Tokyo, Japan; clone TDM-2, 1:1000 dilution), anti-mutant p53 (Abcam, #ab32049, Y5) and anti-γH2AX (ab26350, Abcam).

### Spectral Karyotyping

Exponentially growing cells were incubated with Colcemid (0.1 mg/ml) for 4 h, trypsinized, lysed with hypotonic buffer, and fixed in glacial acetic acid/methanol (1:3). The chromosomes were simultaneously hybridized with 24 combinatorially labeled chromosome painting probes and analyzed using the SD200 spectral bioimaging system (Applied Spectral Imaging Ltd., Migdal Haemek, Israel). Spectral Karyotyping was performed at the Stem cell and Advanced Cell Technologies Unit, Weizmann Institute of Science.

### Comet Assay

DNA damage was measured by comet assay (Comet Assay kit (Catalog #: 4250-050-03), Trevigen, Gaithersburg, MD, USA) per the manufacturer’s instructions. Briefly, cells were detached by trypsinization, washed once, resuspended in ice cold 1X PBS (Ca++ and Mg++ free), counted and resuspended at 1.5 × 10^5^ cells/ml in ice cold 1× PBS (Ca++ and Mg++ free). Cells were then combined with Low melting agarose at a ratio of 1:10 and immediately 50 µl of the suspension was pipetted onto a slide. Slides were then refrigerated for 30 min in the dark, and subsequently immersed in prechilled lysis buffer overnight. The next day, lysis buffer was drained off and slides were immersed in freshly prepared alkaline unwinding solution for 20 min at room temperature. The slides were then placed in an electrophoresis slide tray and electrophoresis was carried out at 20 V with prechilled alkaline electrophoresis solution for 45 min. Subsequently, slides were washed twice with water and once with 70% ethanol for 5 min each. Samples were dried at 37 °C for 10–15 min, stained with SYBR Gold and imaged by epifluorescence microscopy. Imaged comet tails were quantified using the OpenComet plugin (10.1016/j.redox.2013.12.020) with ImageJ software. Graphs were plotted using GraphPad Prism.

### CPD lesions assay

Cells were grown on coverslips and irradiated in 1X PBS buffer with UV‐C using a low‐pressure mercury lamp (TUV 15 W G15T8, Philips) at a dose rate of 0.2 J/m2/s. The UV dose rate was measured using a UVX Radiometer (UVP) equipped with a 254‐nm detector. After irradiation, PBS buffer was removed, and the cells were replenished with fresh culture medium until harvest. Immunofluorescence was performed 24 h after UV irradiation using anti-CPD antibody (CosmoBio, Clone TDM-2) according to the manufacturer’s protocol. Staining was quantified with ImageJ software and plotted using GraphPad Prism.

### Statistical analyses

Results are presented as the mean ± SEM unless stated otherwise. All the experiments were done in triplicates and the number of biological repeats is indicated for each individual experiment. Student’s *t*-test was applied when the data followed a normal distribution *P* values < 0.05 were considered statistically significant. All analyses were performed using GraphPad Prism.

## Results

### Late passage LFS primary dermal fibroblasts undergo WTp53 LOH in culture

**Fig. 1:**
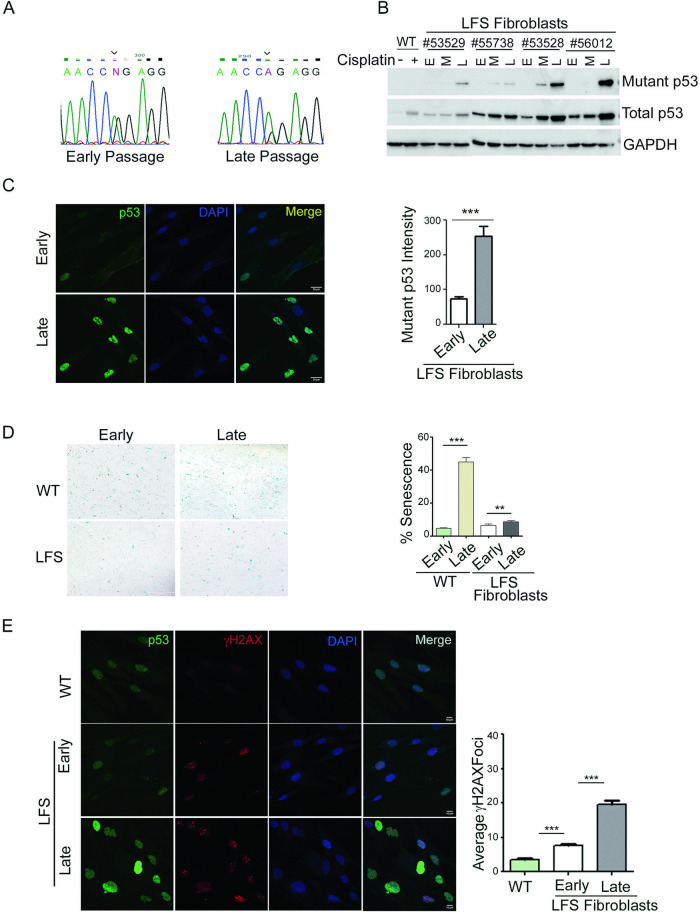
Loss of heterozygosity occurs in late passage LFS fibroblasts. **A** Representative chromatograms showing *TP53* heterozygosity at early passage (left panel) and at late passage (right panel). The adenine peak (arrows) is visible at late passage (*N* = 4). **B** Western blot analysis of lysates from early (E), middle (M), and late (L) passage cells. Total p53 was detected with the DO-1 antibody, and mutp53 was detected with a mutp53–specific antibody. GAPDH was used as a loading control (bottom lane) (*N* = 4). **C** Immunofluorescence analysis of early and late passage LFS fibroblasts. Mutp53–specific antibody (green) was used for LOH confirmation and nuclei were stained with DAPI (blue). (Scale bar, 20 μm.) Quantification of mutp53 staining was done using ImageJ (*N* = 4, *n* > 200). **D** Brightfield microscopic images of LFS fibroblasts undergoing senescence, as detected by β-gal assay; blue indicates senescent cells. Wild-type (WT) p53 fibroblasts at early (P12) and late (P21) and LFS fibroblasts at early and late passage were analyzed (*N* = 4). **E** Immunofluorescence analysis was performed on fibroblasts incubated with FITC-labeled mutp53–specific antibody (green), and yH2AX was detected by red fluorescence. Nuclei were stained with DAPI (blue). (Scale bar, 10 μm.) Quantification of DNA damage marker yH2AX was done using ImageJ (*N* = 4, *n* > 200). *p < 0.05, **p < 0.01, ***p < 0.001. Two-tailed unpaired Student’s *t*-test.

To establish an experimental model of p53 LOH in LFS, we performed genotyping of *TP53* status from mRNA in fibroblast cell lines derived from four LFS patients (Table 5). RNA was isolated from early (9^th^) and late (32^nd^) passages. The p53 DNA binding domain (DBD) cDNA was amplified using flanking primers and subjected to sequencing to distinguish between the mutant and WT p53 mRNA. DNA sequencing showed that late passage cells from all four patients underwent LOH. Early passage cells had a CNG codon at position 248 corresponding to equal peaks of adenine and guanine. Following LOH, the mutant form of *TP53* became dominant with a CAG codon (i.e., arginine was replaced by glutamine in the p53 protein) (Fig. 1A).

**Table 5:** Details of LFS patients from which fibroblast cells are taken.

ID	P53 status	Gender	Age	Cancer
53528	Exon 7: c.743G>A (p.Arg248Gln)	M	45	Multiple myeloma
53529	Exon 7: c.743G>A (p.Arg248Gln)	M	16	Atypical osteochondroma/low grade chondrosarcoma
55738	Exon 7: c.743G>A (p.Arg248Gln)	F	34	Osteosarcoma (humerus)
56012	Exon 7: c.743G>A (p.Arg248Gln)	F	34.3	Rhabdomyosarcoma

To validate *TP53* LOH, we performed western blot analysis. As seen in Fig. 1B (and supplemental material-original data file), mutant p53 (mutp53) protein, assessed by specific antibodies, was expressed in all the late passages in all the cell lines. Moreover, LOH led to an increase in overall p53 levels. It seems, however, that LOH starts to occur already at early stages, probably in the patients, since early passage cells contain a small subpopulation of high mutp53–expressing cells (Fig. 1C). This subpopulation becomes dominant at later passages, probably due to a selective advantage conferred by losing the WT *TP53* allele [27].

### LOH leads to delayed cellular senescence

Senescence is a process by which a cell permanently stops dividing but does not die. Senescent cells usually become enlarged, and their metabolism slows down. Tissue culture shock has been shown to trigger the onset of cellular senescence in culture [28–30]. p53 plays a critical role in cellular responses to stress. Its activation leads to cell cycle arrest, allowing for DNA repair, or triggers cellular senescence or apoptosis, thereby maintaining genome integrity [31, 32]. We therefore examined whether loss of the WT *TP53* allele and mutp53 accumulation affect the onset of senescence. We performed β-Gal staining of cultured healthy control (WT) and LFS fibroblasts in early and late passages. As seen in Fig. 1D, the proportion of senescent cells in late passage LFS fibroblasts was significantly lower than that in WT primary dermal fibroblasts. It is therefore plausible that passaging LFS fibroblasts in culture leads to p53-induced senescence of cells harboring a functional WT *TP53* allele, allowing the small mutp53 homozygous population to take over the culture.

### Loss of the WT *TP53* allele is correlated with increased DNA damage

Human cells are persistently exposed to various chemical and physical agents that have the potential to damage genomic DNA, such as radiation, ultraviolet (UV) light, reactive oxygen species (ROS) and DNA replication errors. p53 plays a central role in maintaining a stable genome in the face of toxic insults, in part by modulating almost all DNA repair processes, including NER, BER, MMR, NHEJ, and HR.

To investigate whether the loss of the WT *TP53* allele is correlated with increased DNA damage, we performed immunofluorescence staining of LFS fibroblasts and monitored yH2AX levels in early passages, as compared to late passages in which the WT *TP53* allele was lost. yH2AX is a DNA damage marker, instigated by double strand DNA breaks. As seen in Fig. 1E, LOH was associated with an increase in yH2AX staining, indicative of increased DNA damage.

### LOH is associated with enhanced metabolism

**Fig. 2:**
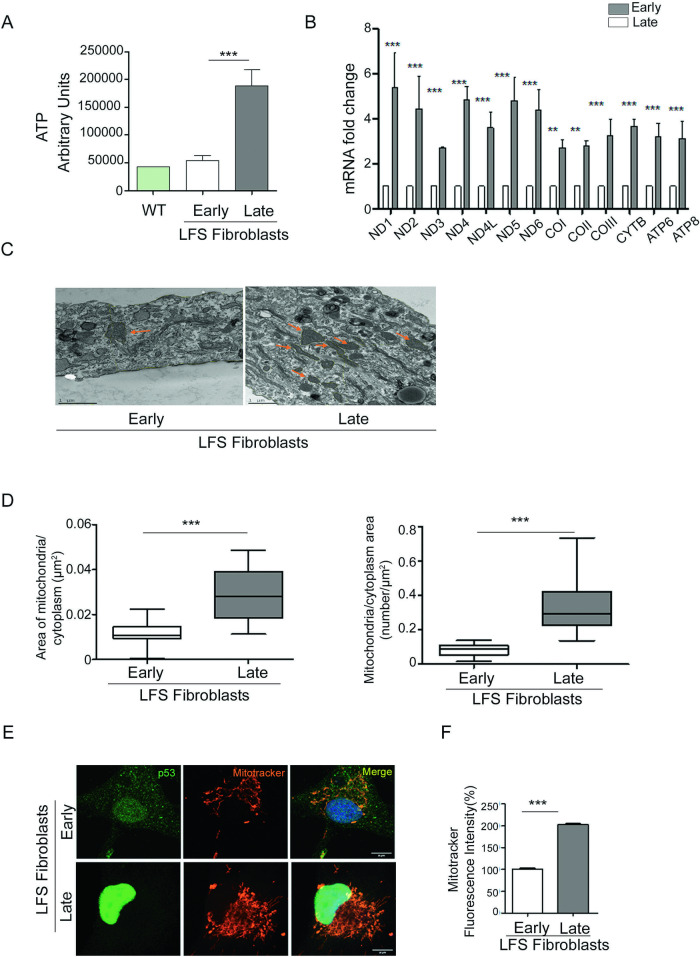
Loss of heterozygosity enhances mitochondrial metabolism. **A** Intracellular ATP levels were measured by ATP Bioluminiscence Assay Kit CLS II on WT fibroblasts, early passage LFS fibroblasts, and late passage LFS fibroblasts (*N* = 4). **B** Human mitochondrial gene expression was measured by RT-qPCR, using specific primers (*N* = 4). **C** Transmission electron microscopy was performed to study mitochondrial size and density at early passage and late passage (mitochondria are shown with orange arrows). Images were taken at 6500× (*N* = 3, number of cells per condition >90) **D** Data were quantified using Image J. **E** Immunofluorescence analysis was performed on early and late passage LFS fibroblasts. Mutp53-specific antibody was used for LOH confirmation, and MitoTracker Red dye was used to label mitochondria Nuclei were stained with DAPI (blue). (Scale bar, 20 μm.) **F** Quantification of mitochondrial fluorescence intensity was performed using ImageJ (*N* = 4, *n* > 150). *p < 0.05, **p < 0.01, ***p < 0.001. Two-tailed unpaired Student’s *t*-test.

Previously, we reported that aggressive cancer cells display increased metabolic rates and mitochondrial mass [3]. Concordantly, we found that cells undergoing LOH display increased metabolism. Specifically, we investigated intracellular ATP content, mitochondrial gene expression, and mitochondrial mass, before and after LOH. Intracellular ATP was significantly higher after p53 LOH as compared to early passage cells (Fig. 2A). Intracellular ATP levels are regulated by mitochondrial genes. We therefore examined the expression profile of all 13 human mitochondrial genes and found a significant upregulation in their expression from early to late passage, consistent with the fact that LOH is accompanied by enhanced mitochondrial metabolism (Fig. 2B).

To further validate these changes, we investigated the mitochondrial mass and density before and after LOH, using transmission electron microscopy. We observed a significant increase in the area ratio of mitochondria/cytoplasm after LOH, as well as an increase in the density ratio of the mitochondria per cytoplasm (Fig. 2C, D). These changes were confirmed also by immunofluorescence, using mutp53-specific antibody and MitoTracker Red. As seen in Fig. 2E, F, the increase in mutp53 levels after LOH was accompanied by increased mitochondrial mass.

### Following LOH, late passage LFS cells facilitate cancer cell migration

Cancer-associated fibroblasts facilitate adjacent tumor cell migration [33]. We therefore assessed the ability of early and late passage LFS fibroblasts to facilitate cancer cell migration. To that end, we incubated U2OS osteosarcoma cells with conditioned medium (CM) from early and late passage LFS fibroblasts. As seen in Fig. S1A, B, CM from late passage LFS fibroblasts that had undergone LOH enhanced tumor cell migration more than CM from early passage cells.

In parallel, we injected the LFS fibroblasts subcutaneously into NOD-SCID mice. As seen in Fig. S1C, the injected fibroblasts did not form tumors, suggesting that they are not tumorigenic on their own. Altogether, our observations suggest that upon LOH and acquisition of high mutp53 levels, LFS fibroblasts may promote tumor progression by enhancing the malignant features of adjacent cancer cells.

### pCAP-250 restricts the increase in mutp53 expression in passaged LFS fibroblasts

**Fig. 3:**
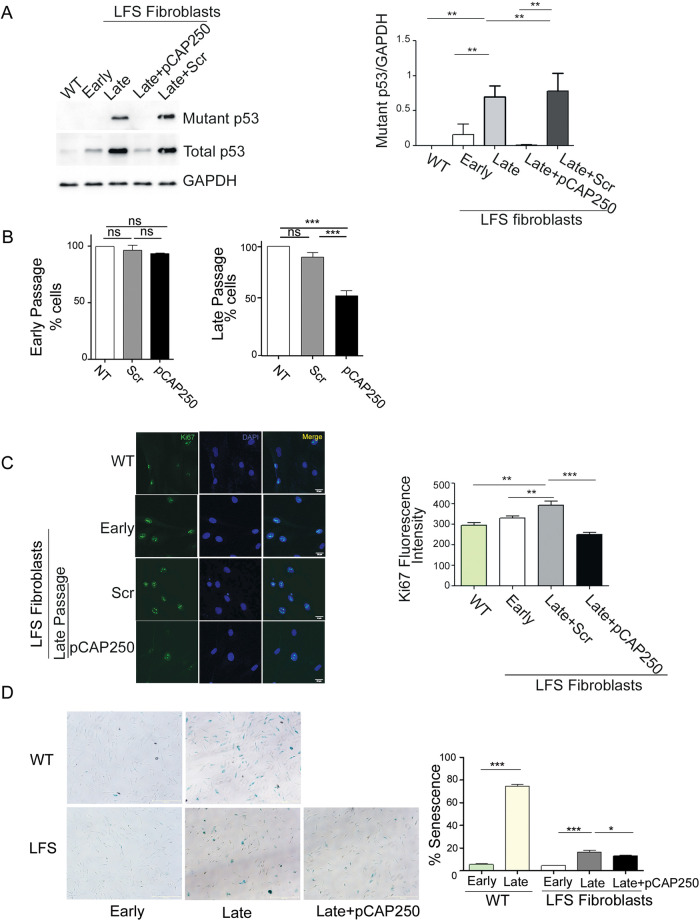
pCAP-250 restricts mutp53 expression and reduces the growth of late passage cells. **A** Western blot analysis of lysates from WT, early passage LFS, late passage LFS, and late passage LFS cells treated with pCAP-250 or with a scrambled peptide. Total p53 was detected with the DO-1 antibody, and mutp53 was detected with a mutp53–specific antibody. GAPDH served as loading control. Data was quantified using ImageJ (*N* = 4). **B** Cells were plated in 10-cm dishes at a density of 100,000 cells/dish and then treated with either pCAP-250 or a scrambled peptide or left untreated as a control (NTC). The cells were counted by brightfield imaging at 4× after 72 h (*N* = 4). **C** Immunofluorescence analysis was performed on nontreated late passage cells and late passage cells treated with pCAP-250. Ki67-specific antibody was labeled with FITC, and the nuclei were stained with DAPI (blue). (Scale bar, 20 μm) Images were taken at 20X and staining was quantified using ImageJ (*N* = 4, *n* > 150). **D** Brightfield microscopic images of cells undergoing senescence, as detected by β-gal assay. Blue represents senescent cells. WT and LFS fibroblasts were imaged at early and late passages, along with late passage LFS cells treated with pCAP-250, and senescent cells were quantified (*N* = 4). *p < 0.05, **p < 0.01, ***p < 0.001, ns not significant. Two-tailed unpaired Student’s -test.

We hypothesized that if we can specifically inhibit the increase in mutp53 in LFS cells, we might be able to inhibit the LOH process and its protumorigenic consequences. With this in mind, we used pCAP-250, a short peptide that reactivates mutp53 by stabilizing the p53 protein structure, leading to activation of p53 target genes and eventually to the death of cancer cells expressing mutp53 [26]. In all experiments, we used 10 µm of pCAP-250 and the cells were maintained with the peptide from early to late passage. As seen in Fig. 3A (and supplemental material-original data file), pCAP-250 completely inhibited mutp53 expression in all the LFS fibroblasts, while scrambled peptide pCAP-704 (Scr) had no effect. Moreover, the expression of the WT p53 target genes p21, PUMA and GDF15, which decreased with LOH, was upregulated in the presence of pCAP-250 (Fig. S1D). This effect was pCAP-250 specific, as treatment of late passage cells with scrambled peptide did not affect significantly the expression of WT p53 target genes (Supplementary Fig. S2). Exposure of pCAP-250-treated late passage cells to cisplatin/or doxorubicin further increased the expression of these genes, supporting the conclusion that the pCAP-250 treated cells possessed functional WT p53, which was absent in the scrambled peptide treated cells (Supplementary Figs. S2 and S3 and supplemental material-original data file).

### pCAP-250 slows down the growth of medium and late-passage LFS cells

To examine if pCAP-250 can modulate the growth rate of LFS fibroblasts, cells were continuously treated from early to late passages with either scrambled peptide or pCAP-250, twice a week. In agreement with the ability of pCAP-250 to reactivate mutp53, there was no effect on the growth of early passage cells, which still express functional wtp53. However, as the cell population accumulated mutp53 with continued passaging, the effect of pCAP-250 became increasingly apparent, leading to lower cell numbers at middle and late passages relative to cultures treated with scrambled peptide (Fig. 3B). This was due to attenuated proliferation, evident by a significant decrease in Ki67-positive cells upon pCAP-250 treatment (Fig. 3C).

### pCAP-250 reduces the senescence of late-passage LFS cells

As shown above, LOH delays the senescence of LFS fibroblasts, as observed also in Fig. 3D. Specifically, whereas in WT fibroblasts the fraction of senescent cells increased from 5.5% in P12 to 75% in P28, in LFS fibroblasts the increase was only from 4.5% to 16.5%. Interestingly, treatment of late passage LFS fibroblasts with pCAP-250 caused a mild but statistically significant reduction in their senescence (Fig. 3D). This effect is specific to pCAP-250 as scrambled control peptide had no effect on the senescence level of LFS late passage cells as well as on WT cells, as shown in Supplementary Figs. S4, S5, and S6A, B. Senescent cells are often more resistant to chemotherapy [34–36], suggesting that pCAP-250 may sensitize such cells to chemotherapy drugs.

### pCAP-250 restores baseline mitochondrial metabolism in late passages

**Fig. 4:**
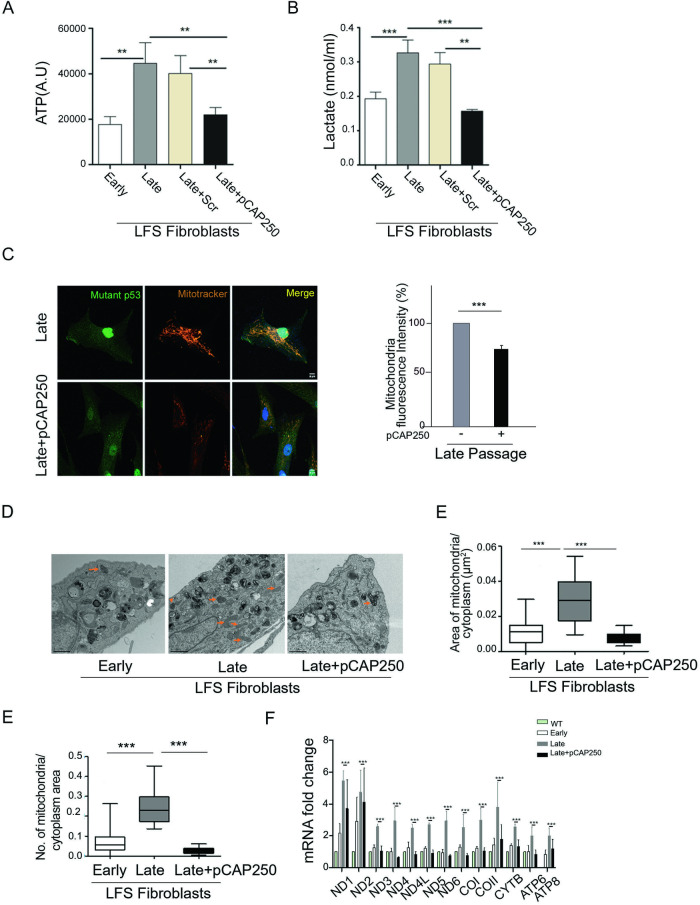
pCAP-250 helps restore mitochondrial metabolism. **A** Intracellular ATP levels were measured by ATP Bioluminiscence Assay Kit CLS II. All fibroblast lines showed enhanced ATP levels after LOH (*N* = 4). **B** Extracellular lactate concentration was measured by Sigma kit. Conditioned medium (50 µL) samples from early passage, late passage, and late passage plus pCAP-250 cells were mixed with master reaction mix and incubated for 30 min. Absorbance was then measured at 570 nm. The X-axis represents concentration (nmol/mL) (*N* = 4). **C** Confocal microscopy was performed on untreated late passage cells and late passage cells treated with pCAP-250. Cells were labeled with mutp53-specific antibody to confirm LOH and MitoTracker Red to label mitochondria. Nuclei were stained with DAPI (blue). (Scale bar, 10 μm.) Quantification was done using ImageJ (*N* = 4, *n* = 100) **D** Electron microscopy was performed to monitor mitochondrial size and density. Images were taken at 6500× at early and late passage and late passage treated with pCAP-250 (mitochondria are indicated by orange arrows) (*N* = 3). **E** Data was quantified using Image J. **F** Human mitochondrial gene expression was measured by RT-qPCR using specific primers (*N* = 4). *p < 0.05, **p < 0.01, ***p < 0.001. Two-tailed unpaired Student’s *t*-test.

As described above, intracellular ATP content levels increased from early to late passage in the LFS fibroblasts. When the same LFS cells were cultivated in the presence of pCAP-250, ATP levels were reduced significantly, indicative of restoration to a non-transformed phenotype (Fig. 4A). Tumor cells secrete extracellular lactate, which can serve as an energy source as part of the Warburg effect [37]. We therefore monitored the extracellular lactate level in serum-free CM of these fibroblasts. As expected, extracellular lactate secretion increased from early to late passages. However, when exposed to pCAP-250, the late passage cells showed a significant reduction in lactate levels (Fig. 4B), which became comparable to those in early passage cells, supporting the effectiveness of pCAP-250 in restoring a normal metabolic phenotype. Scrambled control peptide, at late passage, had no effect on intracellular ATP and lactate secretion levels (Fig. 4A, B).

### pCAP-250 reduces mitochondria number and mitochondria/cytoplasm ratio

As shown above mitochondrial mass, size ratio, and density ratio (i.e., number of mitochondria per cytoplasmic area) increases with passages. Like ATP and lactate levels, pCAP-250 significantly reduced the mitochondrial mass in late passage cells (Fig. 4C). Furthermore, there was a significant reduction in the size ratio of mitochondria/cytoplasm and in the mitochondrial density (Fig. 4D, E). Changes in mitochondrial mass are primarily controlled by mitophagy, a process regulated by Parkin [38, 39]. Loss of WT p53 and gain of mutp53 depletes Parkin in glial tumors [40], and *Parkin* LOH occurs in 33% of colorectal cancers [41], as well as in lung cancer [42] and breast cancer [43, 44]. Therefore, we assessed Parkin levels in early, middle, and late passage LFS fibroblasts treated with pCAP-250. As seen in Supplementary Fig. S7, Parkin levels decreased significantly from early to late passage. Importantly, pCAP-250 not only prevented this decrease but even further increased *Parkin* expression by 50%, compared to early passage cells. Furthermore, with the exception of ND2, all tested human mitochondrial genes were downregulated in late passage cells treated with pCAP-250, as compared to non-treated late passage cells (Fig. 4F).

### pCAP-250 reduces the ability of late passage LFS fibroblasts to promote cancer cell migration

**Fig. 5:**
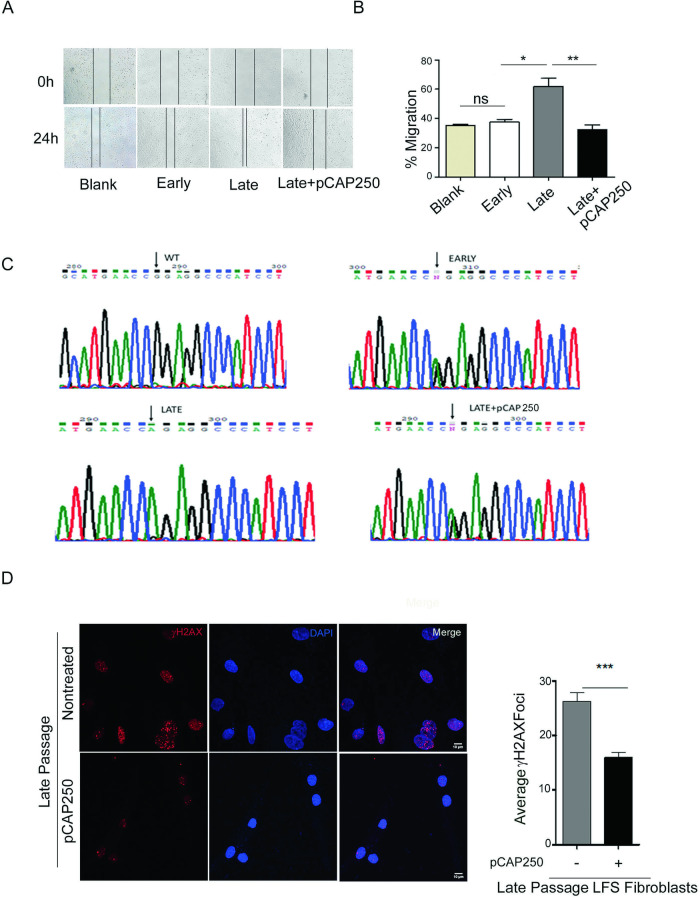
pCAP-250 reduces cancer cell migration and DNA damage and prevents WT *TP53* LOH in late passage LFS cells. **A** Scratch assay was performed on U2OS cells using conditioned medium from LFS fibroblasts at early passage, late passage, and late passage with pCAP-250. Representative images were taken with a brightfield microscope (*N* = 4). **B** Gap distance was quantified for all conditions at 24 h. The Y-axis represents percentage migration, and the X-axis represents passage number. **C** Representative chromatograms depicting the status of the *TP53* gene. Black arrow indicates WT fibroblast (CGG), heterozygous status (CNG) at early passage, mutant only (CAG) at late passage, and heterozygous status (CNG) in late passage treated with pCAP-250 (*N* = 4). **D** Immunofluorescence was performed to monitor γH2AX foci, indicative of DNA damage. DNA damage was measured in late passage cells with or without pCAP-250 treatment. γH2AX was detected by red fluorescence. Nuclei were stained with DAPI (blue) (Scale bar, 10 μm.) Quantification was done using Image J (*N* = 4, *n* > 150). *p < 0.05, **p < 0.01, ***p < 0.001. Two-tailed unpaired Student’s *t*-test.

Since CM from late passage LFS fibroblasts increased cancer cell migration, we examined the effect of pCAP-250 treatment on this activity. As seen in Fig. 5A, B, CM from pCAP-250 treated late passage LFS fibroblasts significantly reduced the migration of U2OS cells, restoring it to control levels. This suggests that pCAP-250 affects the factors secreted by fibroblasts, presumably through its effect on p53.

### Prolonged treatment with pCAP-250 prevents *TP53* LOH in LFS fibroblasts

To examine if pCAP-250 can prevent *TP53* LOH in LFS fibroblasts, we performed genomic DNA sequencing. As shown above, late passage cells show loss of the G nucleotide in the CNG codon, corresponding to the WT *TP53* allele, and retain only the CAG allele that encodes mutp53. Remarkably, continuous exposure of LFS fibroblasts to pCAP-250 caused late passage cells to retain their WT *TP53* allele, as evidenced by the presence of both A and G at this position, maintaining the heterozygous state (Fig. 5C). Treatment with the scrambled peptide did not alter the loss of heterozygosity (LOH) status of late passage cells, as it was comparable to untreated late passage cells (Supplementary Fig. S8). In contrast, when late passage cells were treated with the scrambled peptide, the CAG codon was present, indicating the presence of the mutant p53 encoding sequence. Consequently, pCAP-250 demonstrated the ability to inhibit the occurrence of wild-type TP53 LOH (Fig. 5C and Supplementary Fig. S8).

### pCAP-250 reduces DNA damage

**Fig. 6:**
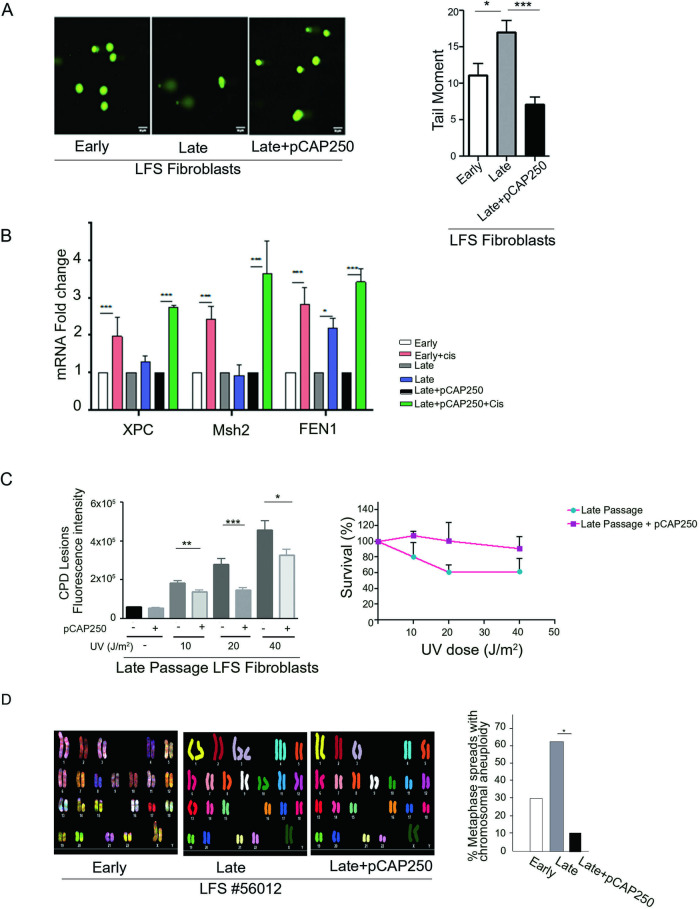
pCAP-250 prevents chromosomal aberrations and repairs DNA damage in late passage LFS fibroblasts. **A** Comet assay was performed on early passage, late passage, and late passage plus pCAP-250 cultures. Data was acquired using epifluorescence imaging (Scale bar, 50 μm.) and Tail length was quantified using ImageJ (*N* = 4, *n* > 200). **B** RT-qPCR was performed on early passage cells, late passage cells and late passage cells treated with pCAP-250, to quantify the expression of genes encoding the DNA repair enzymes XPC, MSH2 and FEN1, which are regulated by WT p53. Cisplatin served as a positive control to induce the expression of these genes by WTp53 activation (*N* = 4). **C** CPD lesions were detected in late passage cells 53528 treated with increasing doses of UV radiation. The cells were incubated in culture medium for 24 h, and lesions were counted and quantified by ImageJ (*n* = 50). **D** Spectral karyotyping was performed on LFS early passage, late passage, and late passage cells treated with pCAP-250 (*n* = 10). Percentages of cells with chromosomal aberrations were calculated. *p < 0.05, **p < 0.01, ***p < 0.001, ns not significant. Two-tailed unpaired Student’s *t*-test.

To examine whether pCAP-250 affects DNA repair and overall genomic stability of LFS fibroblasts, we performed γH2AX staining. As seen in Fig. 5D, the staining intensity of γH2AX significantly increases with the passaging of LFS cells in parallel to the LOH, becoming 2.5-fold higher at late passage than in early passage. Likewise, prolonged pCAP-250 treatment significantly reduced γH2AX levels in late passages of LFS cells (Fig. 5D). Similar conclusions were obtained when we evaluated DNA damage by the comet assay (Fig. 6A). As expected, late passage cells exhibited higher DNA damage compared to early passage. Reassuringly, prolonged treatment of late passage cells with pCAP-250 significantly lowered the levels of DNA damage, even when compared to early passage cells. Presumably, this may be due to reactivation of the mutp53 and enhanced activation of DNA repair by two functional p53 alleles.

Since reactivation of mutp53 by pCAP-250 entails transactivation of WT p53 target genes, we next analyzed the expression of the DNA repair-related genes XPC1, Msh2 and Fen1, reported as transcriptional targets of p53. As seen in Fig. 6B, all 3 genes were responsive to cisplatin in early passage cells but less so in late passages. Remarkably, treatment with pCAP-250 significantly upregulated the mRNA levels of these genes following DNA damage, even beyond that of early passage cells. Thus pCAP-250 may prevent DNA damage, at least in part, via increasing the induction of DNA repair genes by the reactivated mutp53. Interestingly, this DNA repair effect of pCAP-250 was more pronounced in the presence of doxorubicin. Treatment of cells with doxorubicin causes DNA damage, resulting in cell death at high doses of the drug. However, pCAP-250 elicited protection against doxorubicin cytotoxicity, as evident from Supplementary Fig. S9.

### pCAP-250 restores genomic stability

The XPC protein helps repair UV-induced damage by nucleotide excision repair. Therefore, we assessed the ability of pCAP-250-treated cells to repair UV-induced damage. To that end, we quantified cyclopyrimidine dimer (CPD) lesions in LFS fibroblasts exposed to different UV radiation doses. As seen in Fig. 6C, the number of CPD lesions increased with increasing UV doses. Importantly, the number of lesions was significantly reduced upon pCAP-250 treatment. Furthermore, pCAP-250-treated late passage LFS fibroblasts fared better after UV radiation-induced DNA damage than their non-treated counterparts (Supplementary Fig. S10).

To evaluate the overall effect of pCAP-250 on the genomic stability of LFS fibroblasts, about 10 randomly chosen fibroblasts from early passage, late passage and late passage + pCAP-250 were subjected to spectral karyotyping (SKY) analysis. Stained chromosomes from representative cells are shown in the left panel of Fig. 6D, while the right panel shows quantification of chromosomal aberrations in each group. As seen about 30% of LFS fibroblasts exhibited chromosomal aberrations already at early passage. As expected, the proportion of cells with genomic instability rose two-fold to 60% in late passages. Remarkably, prolonged treatment with pCAP-250 caused a significant decrease in the proportion of cells with chromosomal aberrations, down to 10%, even much lower than early passage cells. This activity of pCAP-250 in “normal” non-transformed cells is of particular interest: if indeed pCAP-250 is capable of maintaining genomic stability also in epithelial cells, it may open the possibility to use pCAPs as a preventive treatment, in LFS in particular and in cancer in general.

## Discussion

LFS patients develop a broad spectrum of tumors, most of which exhibit loss of the WT *P53* allele, considered a primary event that precedes cancer progression [45]. It is therefore plausible that prevention or delay of LOH might prevent the onset of cancer in *TP53* pathogenic variant carriers. We show here that primary LFS fibroblasts contain a rare subpopulation of cells that have undergone spontaneous LOH and express high level of mutp53. As primary cells are transferred to culture, they are exposed to high oxygen and other ex-vivo stress factors that cause culture shock. Cells that have a functional WT *TP53* allele respond by undergoing growth arrest and senescence, which allows the homozygous mutp53 subpopulation that remains proliferative to take over the cultured population in the course of 10-20 passages.

Importantly, we now show that treatment of LFS fibroblasts with the mutp53-reactivating peptide pCAP-250 attenuates the acquisition of LOH, and rectifies the aberrant features that accompany the emergence of LOH, including changes in mitochondrial metabolism. This effect of pCAP-250 may be partially attributed to increased levels of Parkin, a transcriptional target of WT p53. High mitochondrial metabolism is also associated with fast cellular growth [46]. Concordantly, cells treated with pCAP-250 grew slower than non-treated cells.

Secreted factors from CM of late passage fibroblasts, but not from fibroblasts treated with pCAP-250, enhanced the migration of cancer cells. Presumably, the LFS fibroblasts after LOH behave similarly to cancer-associated fibroblasts, which secrete various cytokines and chemokines that facilitate cancer cell proliferation and migration, and this is prevented by treatment with pCAP-250.

*TP53* is of critical importance for maintaining genomic stability [47]. Indeed, we show that fibroblasts that have undergone LOH accumulate more DNA damage than heterozygous fibroblasts. In cells with functional wtp53, high DNA damage can elicit senescence or apoptosis [48]. In contrast, late passage fibroblasts that have lost wtp53 fail to undergo efficient senescence. However, treatment with pCAP-250 restored DNA damage to levels that are even below those of early passage fibroblasts. Furthermore, pCAP-250 treatment significantly elevated the expression of transcriptional targets of p53 involved in DNA repair (e.g., FEN1, Msh2, and XPC). pCAP-250 also caused a significant upregulation of DNA repair activity of UV radiation-induced CPD lesions. The reduction in DNA damage can be attributed to increased DNA repair, since no significant cell death was observed with pCAP-250 treatment.

Remarkably, prolonged treatment with pCAP-250 caused a significant decrease in the proportion of cells with chromosomal aberrations, raising hope that peptides such as pCAP-250 may prove efficacious in cancer prevention. Interestingly, prolonged treatment with pCAP-250 significantly reduced the proportion of senescent cells in late passage LFS cells. Since DNA damage has a major influence on the onset of senescence, the ability of pCAP-250 to restrict DNA damage in LFS cells might account for the reduced senescence of the late passage LFS cells.

This is the first report in which a peptide was shown to inhibit LOH in LFS patient-derived cells. Together, our findings suggest that pCAP-250 inhibits LOH and reactivates mutp53, leading to activation of p53 transcriptional targets, enhanced DNA repair, enhanced genomic stability, reduced mitochondrial metabolism, decrease in senescence phenotype and attenuated migration of cancer cells. Thus, pCAP-250 merits further consideration as a means to prevent LOH in LFS mutation carriers and to improve combination chemotherapy for tumors harboring *TP53* missense mutations.

### Supplementary information

Supplementary Data File

Characterization of LFS fibroblasts.

Effect of pCAP-250 and control scrambled peptide in combination with doxorubicin on the expression of WTp53 target genes.

Effect of pCAP-250 in combination with doxorubicin on p53 conformation and levels of the WTp53 target p21.

Effect of pCAP-250 and control scrambled peptide on senescence of LFS fibroblasts in combination with doxorubicin.

Effect of pCAP-250 and control scrambled peptide on senescence of normal fibroblasts in combination with doxorubicin.

Quantification of effect of pCAP-250 and control scrambled peptide on senescence in combination with doxorubicin.

Relative expression of Parkin.

Chromatograms depicting the status of the TP53 gene in all LFS patient samples treated with scrambled peptide and pCAP-250.

Effect of two active pCAPs and scrambled control peptide on the viability of early and late passage LFS cells in response to doxorubicin.

Representative images of UV treated cells at early, late passage and late passage cells treated with pCAP-250.

Original Data Files_Western Blots
