# Supplementary material for: Mutant p53 reactivation restricts the protumorigenic consequences of wild type p53 loss of heterozygosity in Li-Fraumeni syndrome patient-derived fibroblasts
Source: Cell Death Differ. 2024 May 14;31(7):855–67. doi: 10.1038/s41418-024-01307-4 (PMC11239894; doi:10.1038/s41418-024-01307-4)
Supplement: Supplementary file 1 — Supplementary Data File [file 41418_2024_1307_MOESM1_ESM.docx]

**Supplementary Figure Legends**

**Figure S1. Characterization of LFS fibroblasts.** **A)** Scratch assay was performed on U2OS cells using conditioned media from LFS fibroblasts at early or passage. Representative images were taken with a Brightfield microscope at 4x (N=4). **B)** Data was quantified with Graph Pad Prism. **C)** Late passage fibroblasts derived from LFS patients were subcutaneously injected into the flanks of NOD-SCID mice. PC9 cells served as positive control (n=5 per cell line). **D)** RT-qPCR was performed on early passage cells, late passage cells and late passage cells treated with pCAP-250, to quantify the expression of genes regulated by WT p53 with or without cisplatin treatment for 4 hours (N=4).

**Figure S2. Effect of pCAP-250 and control scrambled peptide in combination with doxorubicin on the expression of WTp53 target genes.** Early and late passage fibroblasts derived from LFS patients were treated with either pCAP-250 or a control scrambled peptide (pCAP-704) (10µM) in combination with doxorubicin (0.2µg/ml) for 24h. Gene expression was measured by RT-qPCR using specific primers for 4 representative p53 target genes.

**Figure S3. Effect of pCAP-250 in combination with doxorubicin on p53 conformation and levels of the WTp53 target p21.** Western blot was performed on lysates from early passage and late passage cells treated with pCAP-250, using the DO-1 antibody to detect total p53 and a mutant p53–specific antibody to detect only mutant p53. GAPDH was used as a loading control.

**Figure S4. Effect of pCAP-250 and control scrambled peptide on senescence of LFS fibroblasts in combination with doxorubicin.** Cells at the specified passages were cultured either with or without pCAP-250 for three weeks, with the addition of doxorubicin (0.2µg/ml) for one week, followed by β-gal staining using a commercial kit (Sigma). Representative images of senescence-associated β-Gal staining of LFS fibroblasts at early and late passages.

**Figure S5. Effect of pCAP-250 and control scrambled peptide on senescence of normal fibroblasts** **in combination with doxorubicin.** Representative images of senescence-associated β-Gal staining of normal fibroblasts at early and late passages.

**Figure S6. Quantification of** **effect of pCAP-250 and control scrambled peptide on senescence in combination with doxorubicin.** Quantification of senescence-associated β-Gal staining in presence and absence of doxorubicin for **A)** LFS fibroblasts at early and late passages **B)** normal fibroblasts at early and late passages.

**Figure S7. Relative expression of Parkin.** Parkin gene expression measured by RT-qPCR in early and late passage cells and late passage cells treated with pCAP-250 (N=4).

**Figure S8. Chromatograms depicting the status of the *TP53* gene in all LFS patient samples treated with scrambled peptide and pCAP-250**. Red arrow indicates LFS fibroblast heterozygous status (CNG) at early passage, mutant only (CAG) at late passage, mutant only (CAG) in late passage treated with scrambled peptide and heterozygous status (CNG) at late passage cells treated with pCAP-250.

**Figure S9. Effect of two active pCAPs and scrambled control peptide on the viability of early (left) and late (right) passage LFS cells in response to doxorubicin.** Cells were seeded into 96-well plates and treated on the subsequent day with the specified peptides (10µM), along with escalating concentrations of doxorubicin. After 5 days, the cells were fixed, stained, and the number of viable cells was quantified and normalized to the non-treated group (set at 100%).

**Figure S10. Representative images of cells at early and late passage and late passage cells treated with pCAP-250**. Cells were grown on coverslips and irradiated with UV‐C in 1X PBS buffer using a low‐pressure mercury lamp (TUV 15W G15T8, Philips) at a dose rate of 0.2 J/m2/s. Brightfield images were taken at 10x (N=2).
