# Supplementary figures and images for: Mutant p53 reactivation restricts the protumorigenic consequences of wild type p53 loss of heterozygosity in Li-Fraumeni syndrome patient-derived fibroblasts
Source: Cell Death Differ. 2024 May 14;31(7):855–67. doi: 10.1038/s41418-024-01307-4 (PMC11239894; doi:10.1038/s41418-024-01307-4)

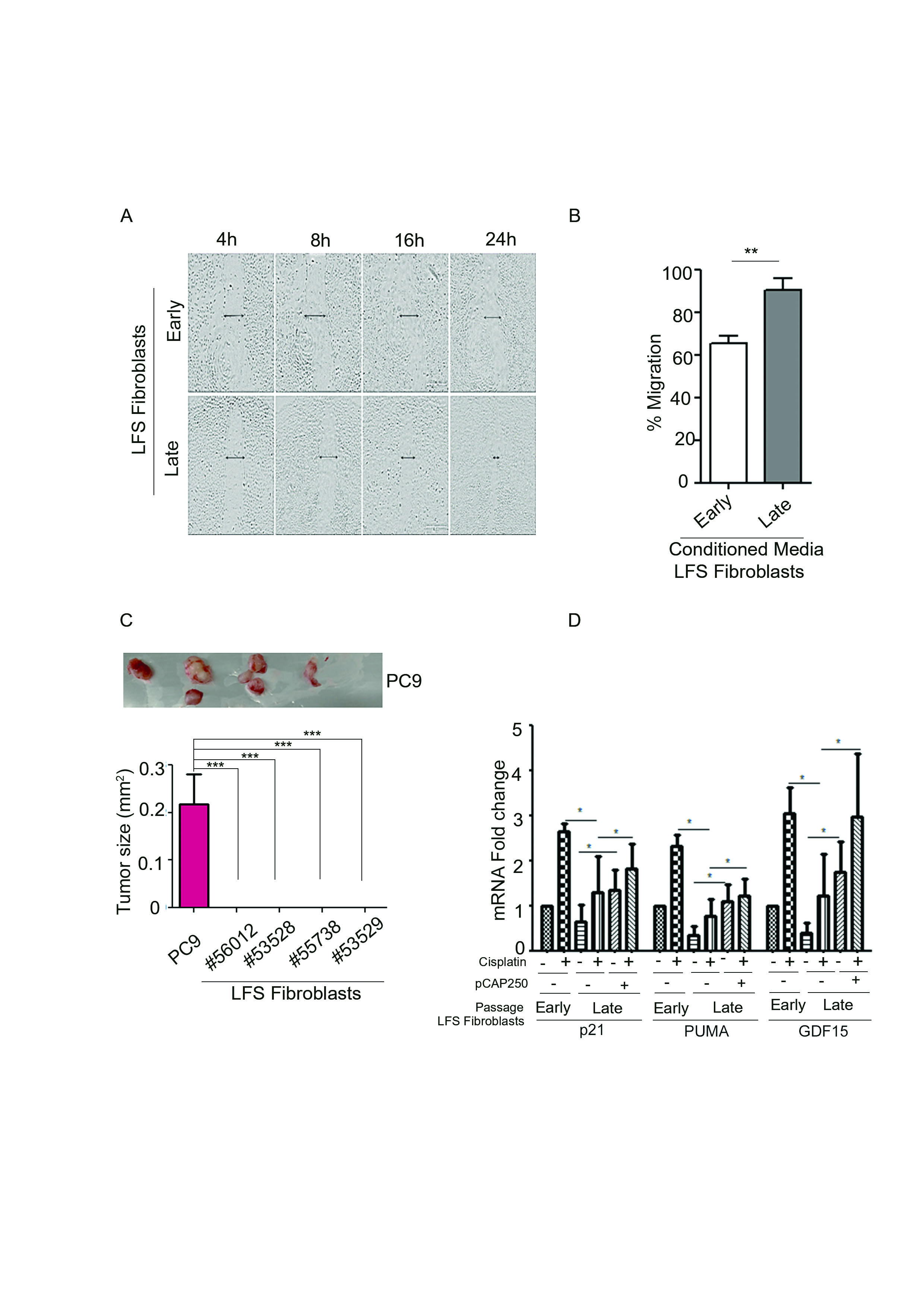

Supplement: Supplementary file 2 — Characterization of LFS fibroblasts. [file 41418_2024_1307_MOESM2_ESM.jpg]

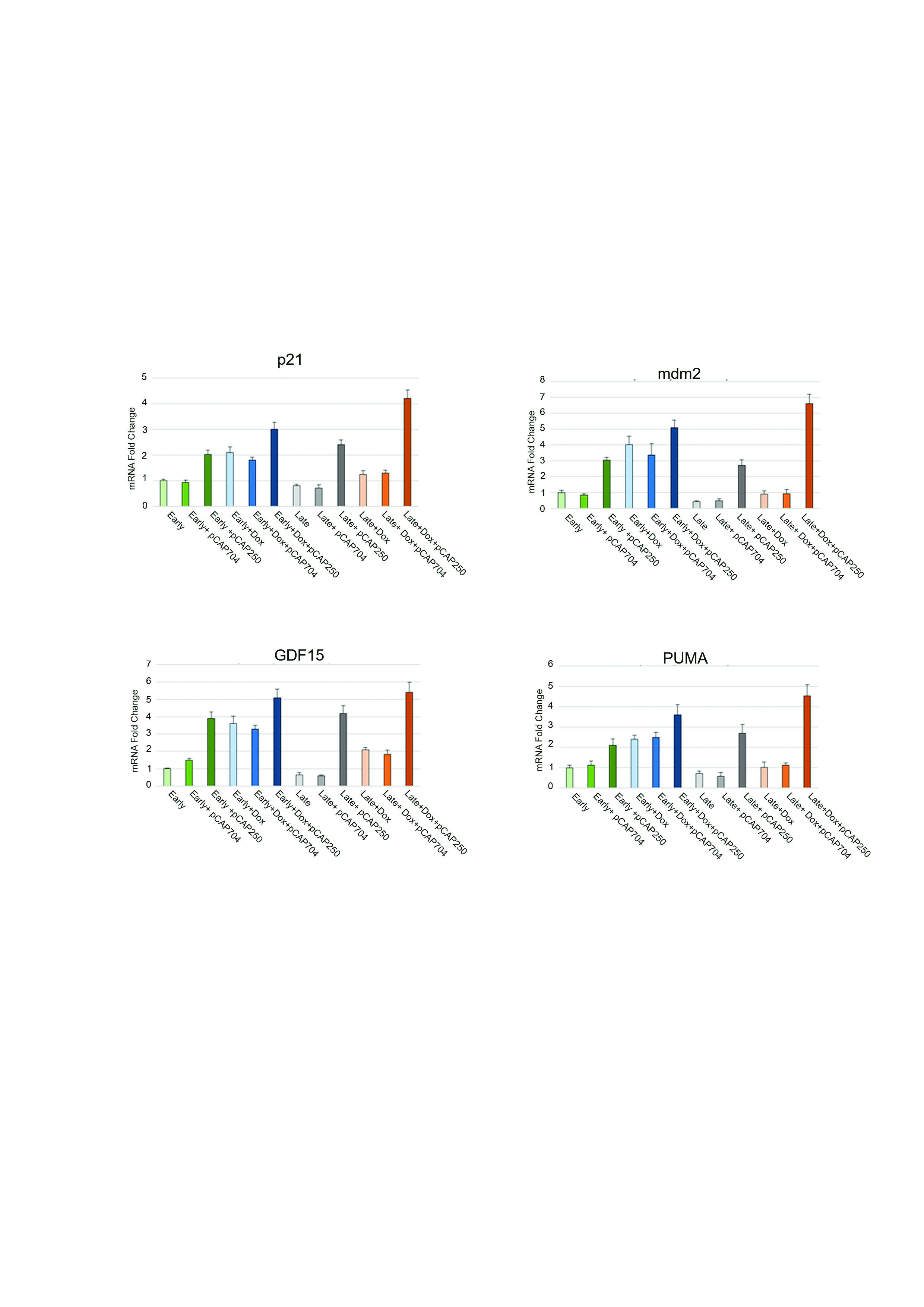

Supplement: Supplementary file 3 — Effect of pCAP-250 and control scrambled peptide in combination with doxorubicin on the expression of WTp53 target genes. [file 41418_2024_1307_MOESM3_ESM.jpg]

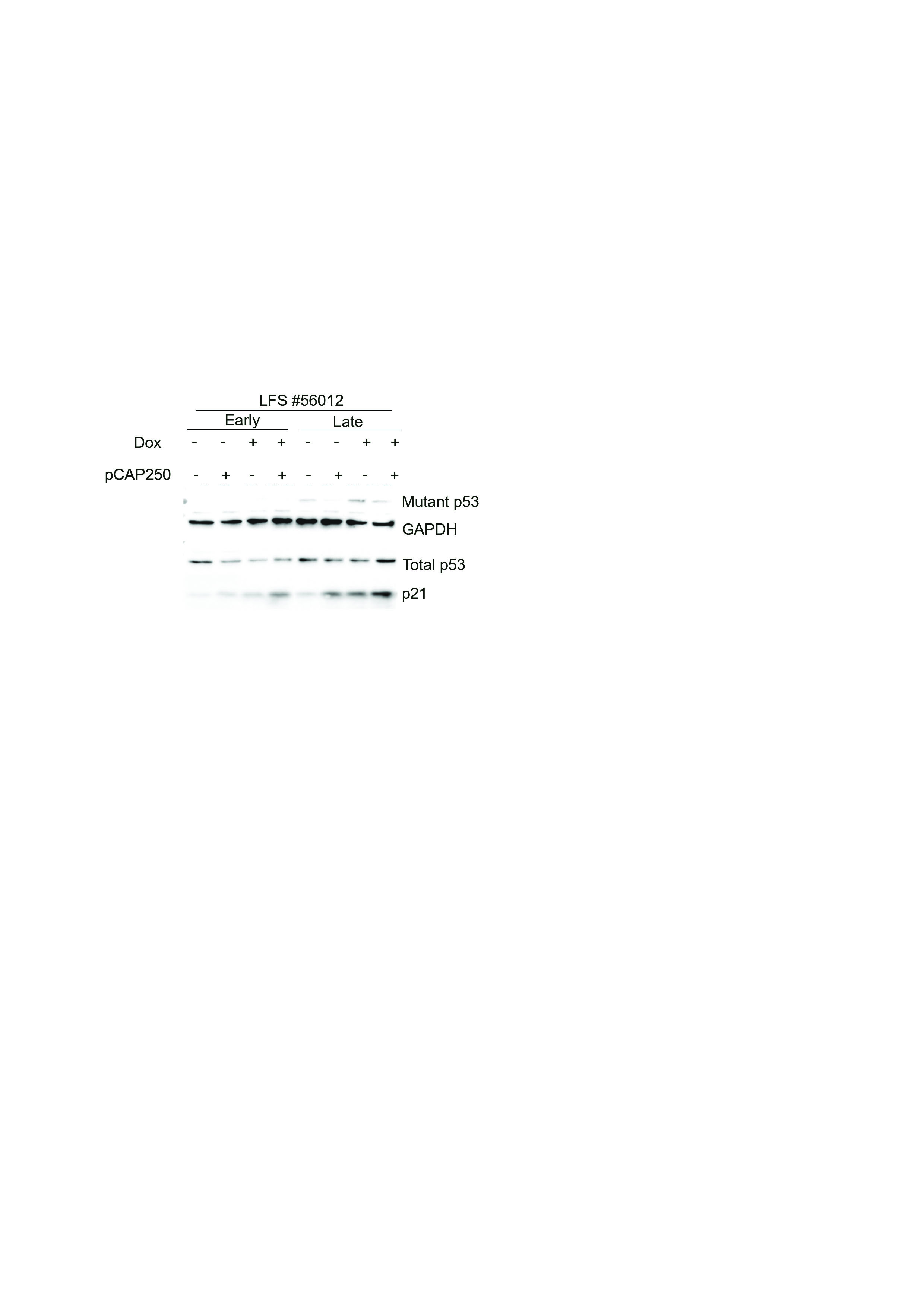

Supplement: Supplementary file 4 — Effect of pCAP-250 in combination with doxorubicin on p53 conformation and levels of the WTp53 target p21. [file 41418_2024_1307_MOESM4_ESM.jpg]

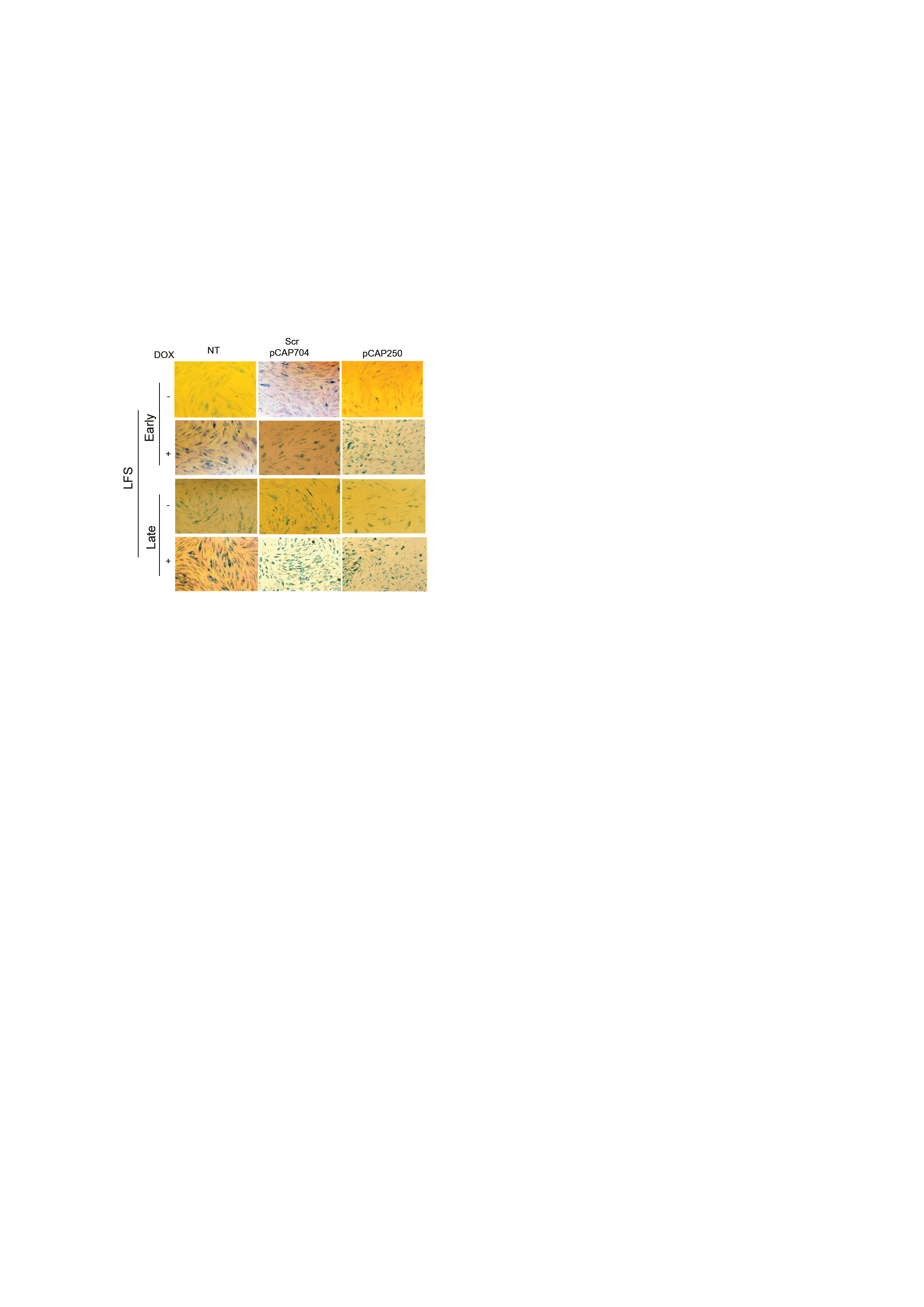

Supplement: Supplementary file 5 — Effect of pCAP-250 and control scrambled peptide on senescence of LFS fibroblasts in combination with doxorubicin. [file 41418_2024_1307_MOESM5_ESM.jpg]

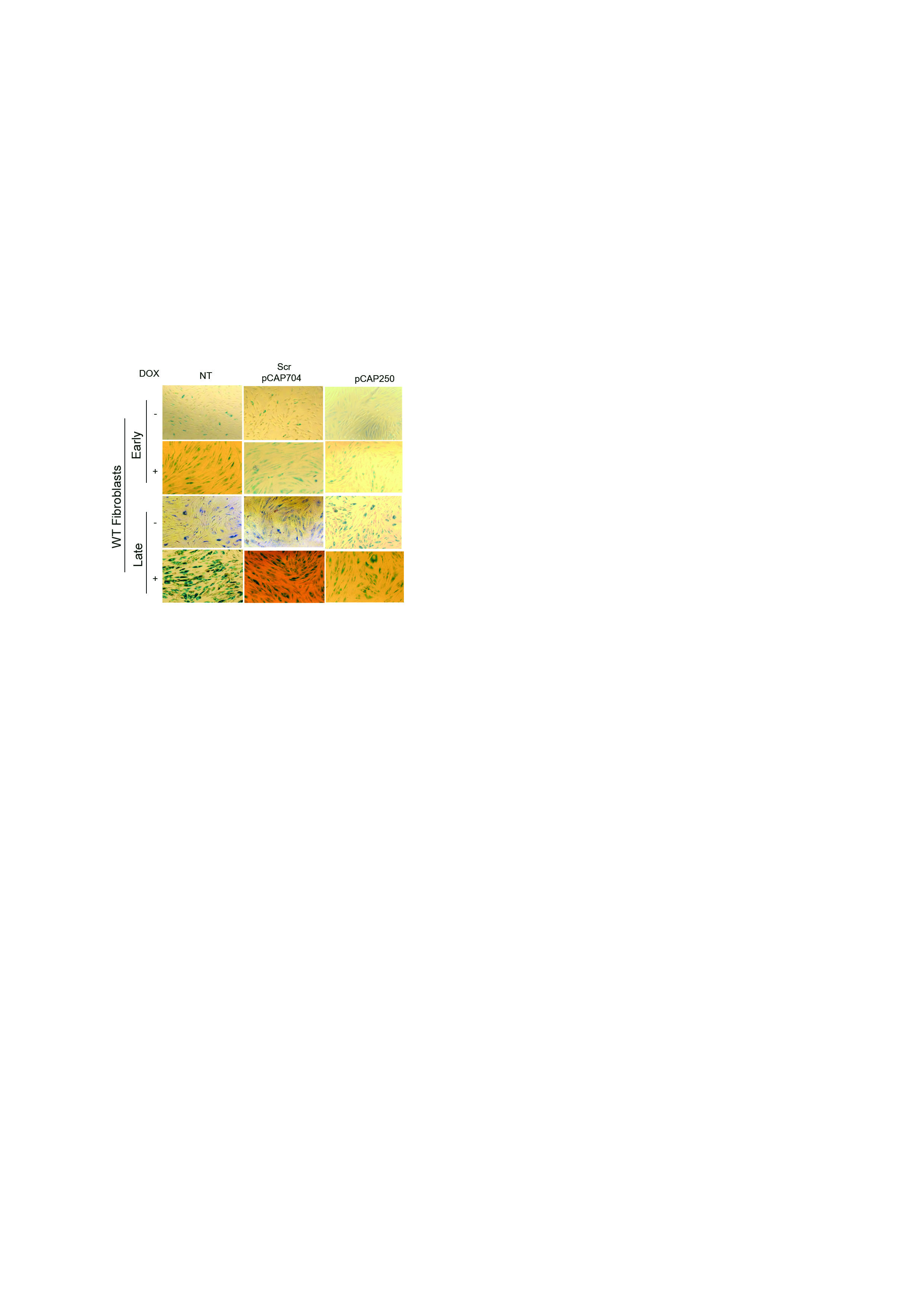

Supplement: Supplementary file 6 — Effect of pCAP-250 and control scrambled peptide on senescence of normal fibroblasts in combination with doxorubicin. [file 41418_2024_1307_MOESM6_ESM.jpg]

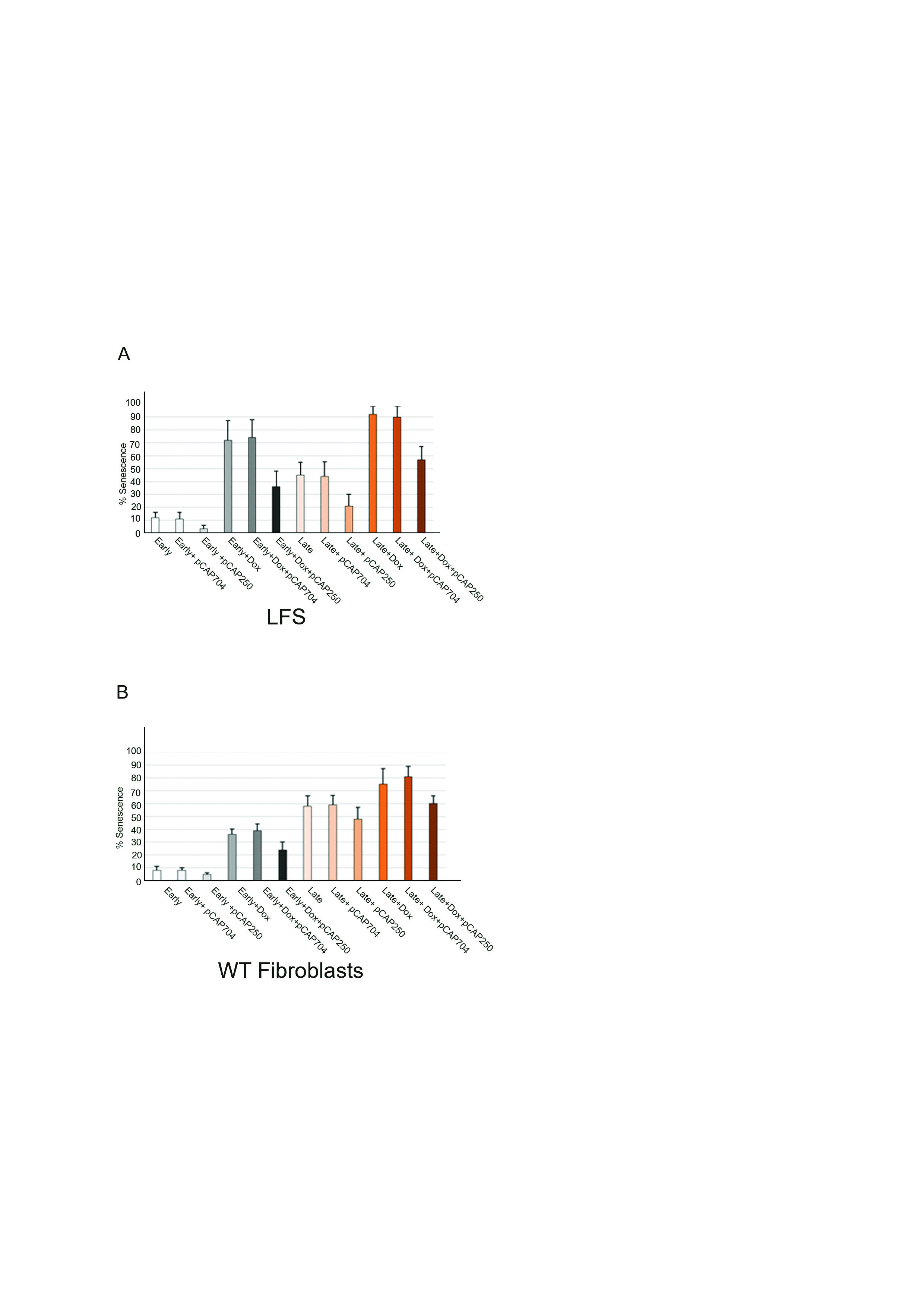

Supplement: Supplementary file 7 — Quantification of effect of pCAP-250 and control scrambled peptide on senescence in combination with doxorubicin. [file 41418_2024_1307_MOESM7_ESM.jpg]

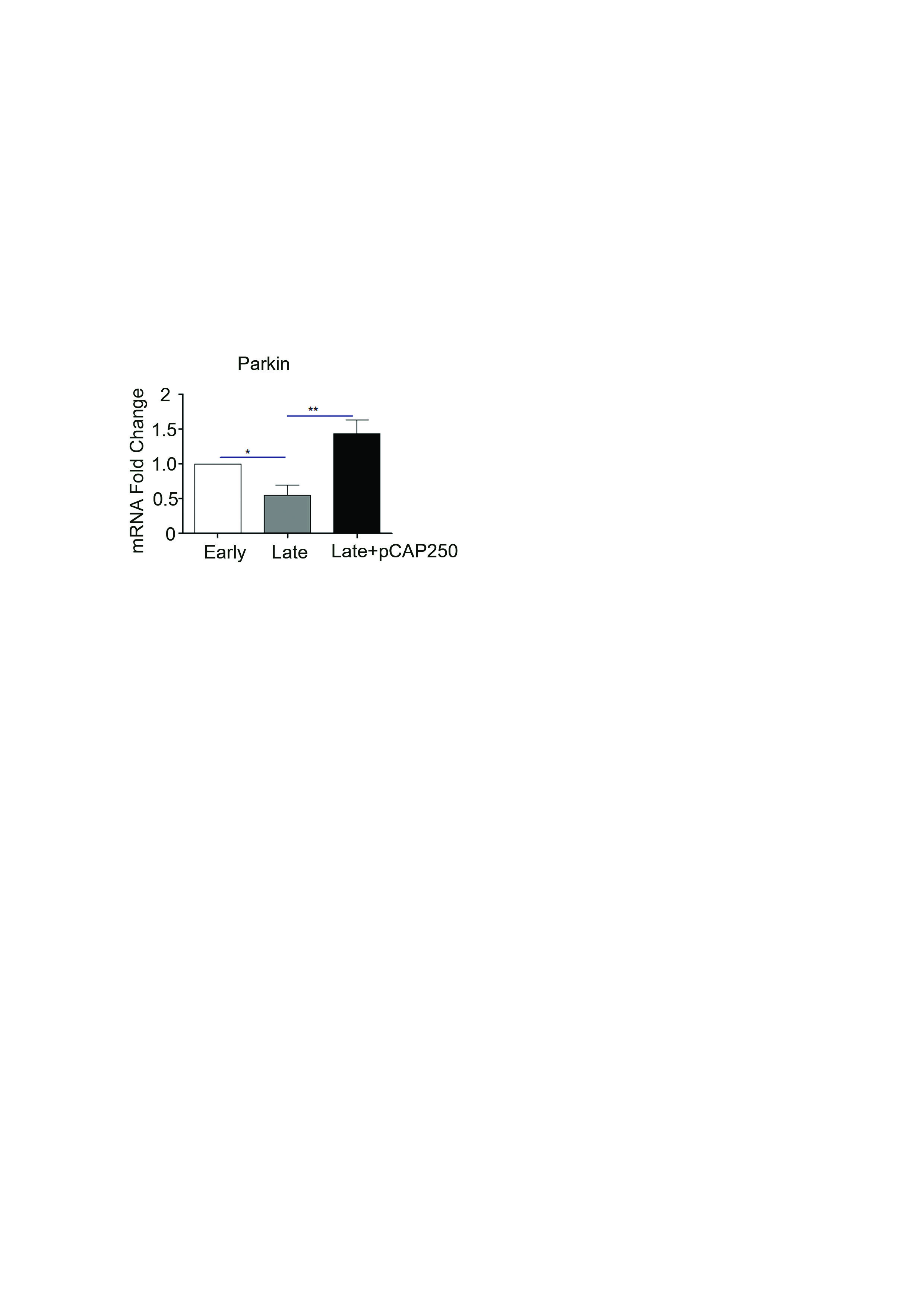

Supplement: Supplementary file 8 — Relative expression of Parkin. [file 41418_2024_1307_MOESM8_ESM.jpg]

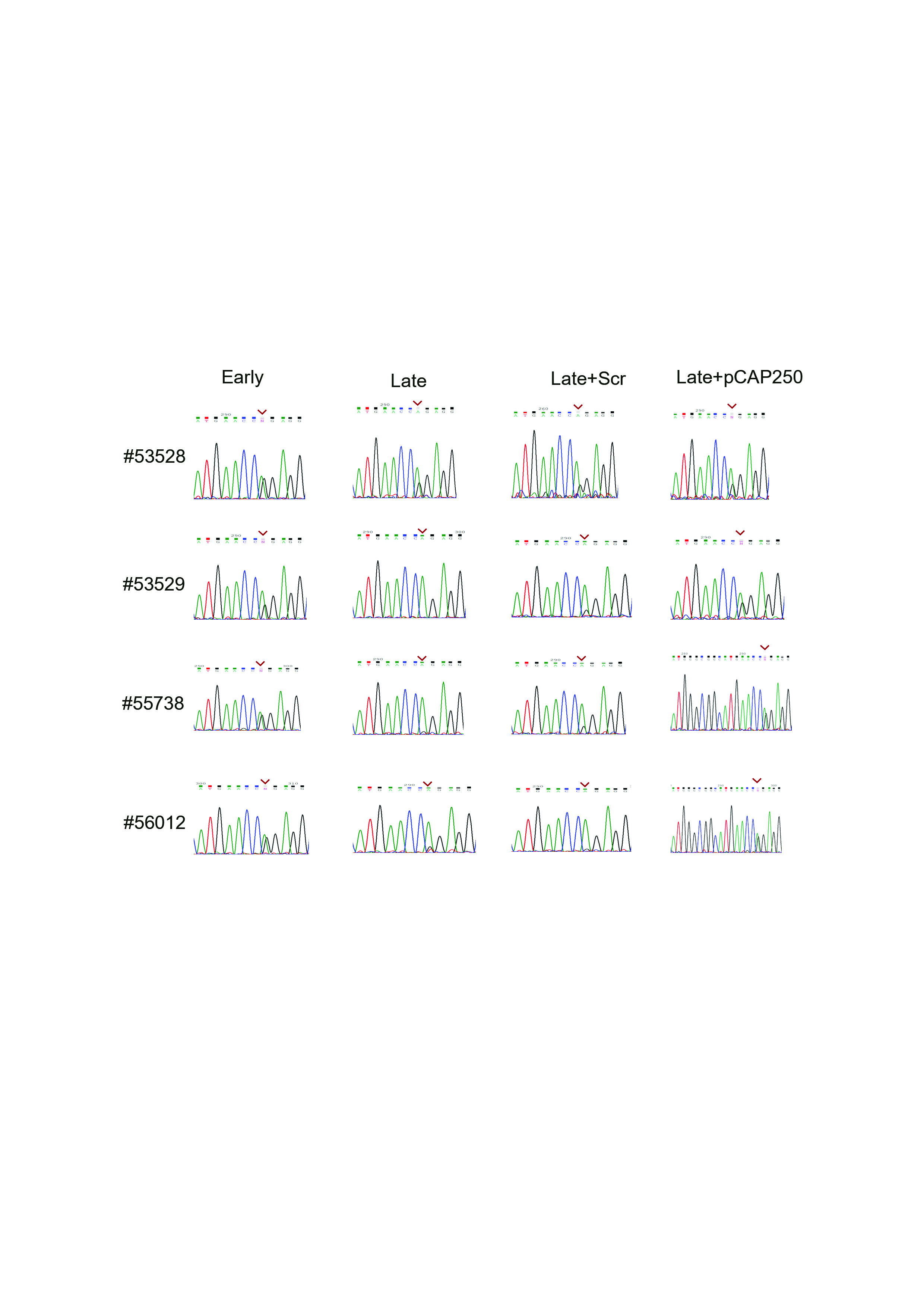

Supplement: Supplementary file 9 — Chromatograms depicting the status of the TP53 gene in all LFS patient samples treated with scrambled peptide and pCAP-250. [file 41418_2024_1307_MOESM9_ESM.jpg]

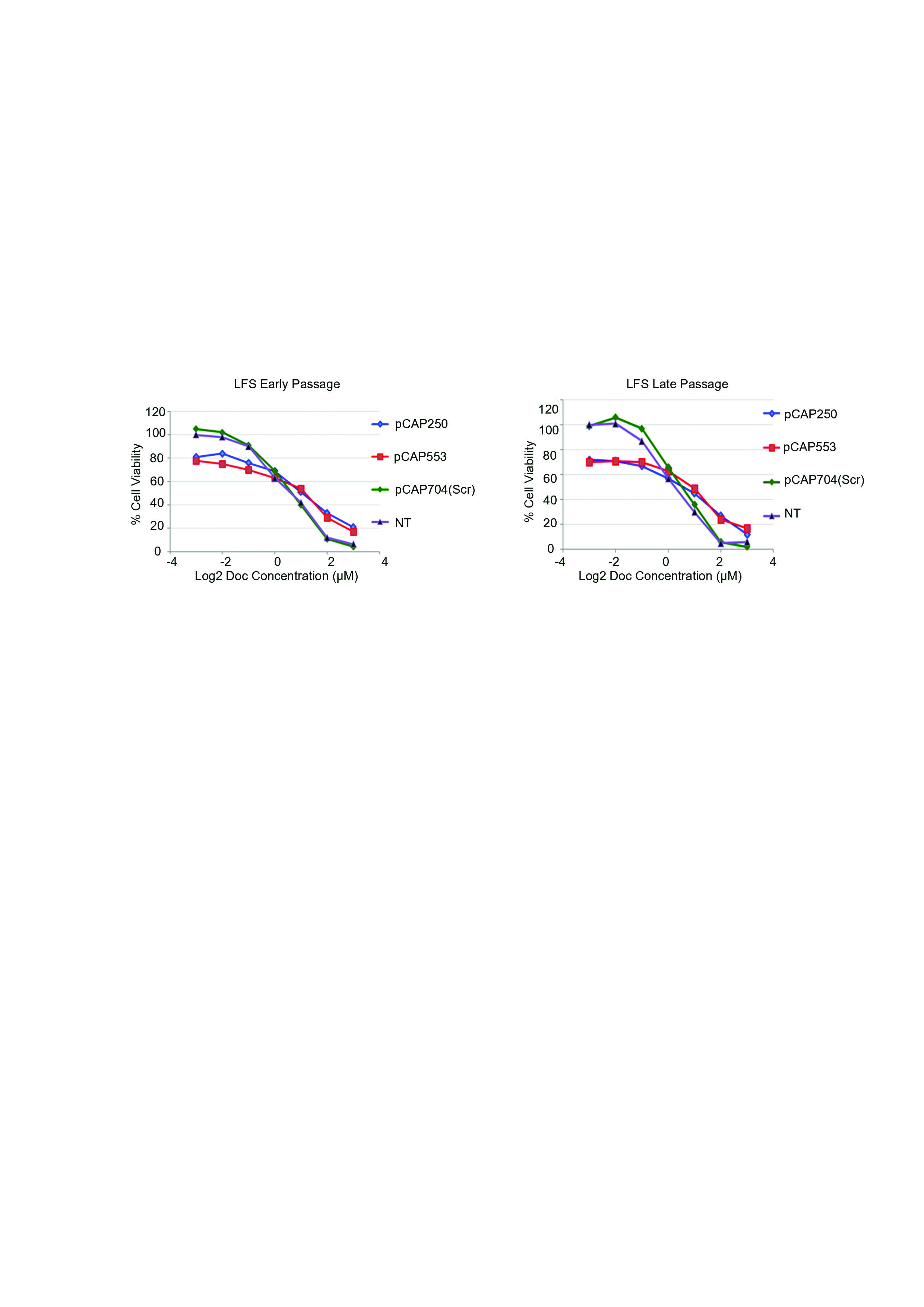

Supplement: Supplementary file 10 — Effect of two active pCAPs and scrambled control peptide on the viability of early and late passage LFS cells in response to doxorubicin. [file 41418_2024_1307_MOESM10_ESM.jpg]

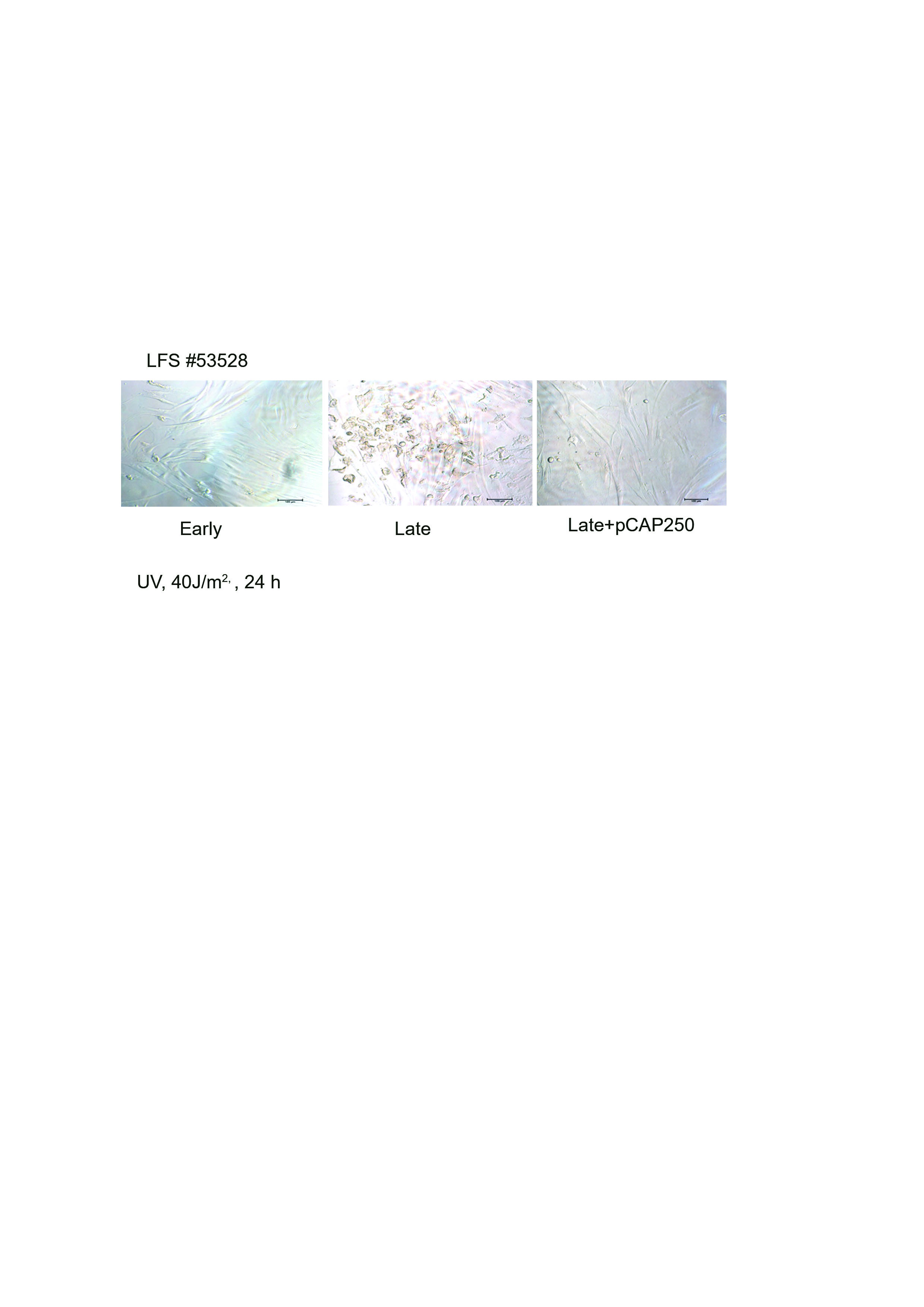

Supplement: Supplementary file 11 — Representative images of UV treated cells at early, late passage and late passage cells treated with pCAP-250. [file 41418_2024_1307_MOESM11_ESM.jpg]

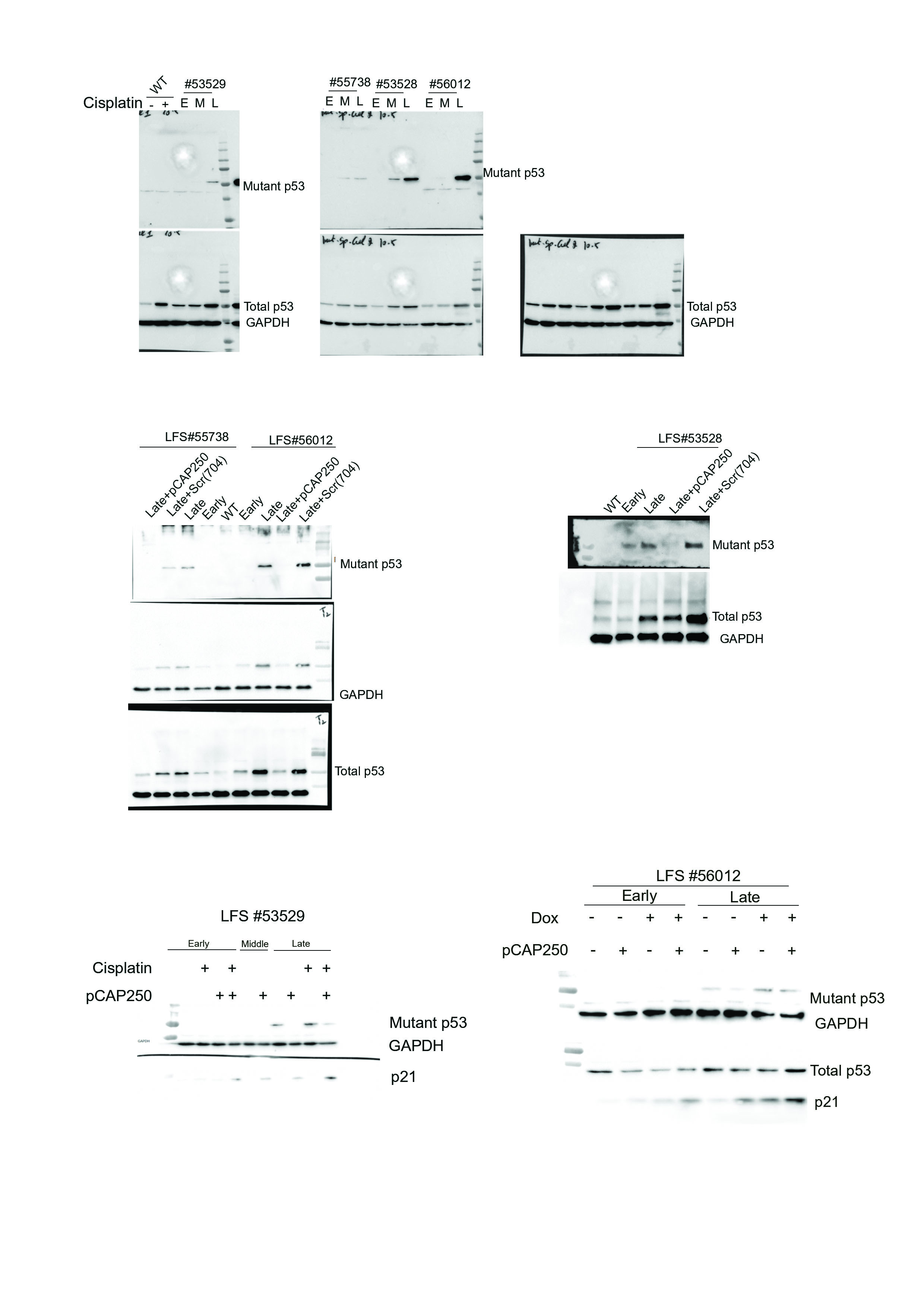

Supplement: Supplementary file 12 — Original Data Files_Western Blots [file 41418_2024_1307_MOESM12_ESM.jpg]
